# Natural Killer Response and Lipo-Metabolic Profile in Adults with Low HDL-Cholesterol and Mild Hypercholesterolemia: Beneficial Effects of Artichoke Leaf Extract Supplementation

**DOI:** 10.1155/2019/2069701

**Published:** 2019-01-06

**Authors:** M. Rondanelli, A. M. Castellazzi, A. Riva, P. Allegrini, M. A. Faliva, G. Peroni, M. Naso, M. Nichetti, C. Tagliacarne, C. Valsecchi, T. Fazia, S. Perna, F. Graziano, M. Grassi, L. Bernardinelli

**Affiliations:** ^1^IRCCS Mondino Foundation, Italy; ^2^Department of Public Health, Experimental and Forensic Medicine, University of Pavia, Italy; ^3^Department of Clinical Surgical Diagnostic and Pediatric Sciences, University of Pavia, Pavia, Italy; ^4^Research and Development Unit, Indena, Milan, Italy; ^5^Endocrinology and Nutrition Unit, Section of Human Nutrition, ASP Pavia, University of Pavia, 27100, Pavia, Italy; ^6^Department of Brain and Behavioral Science, University of Pavia, Pavia, Italy; ^7^Department of Biology, College of Science, University of Bahrain, Sakhir Campus, P.O. Box 32038, Bahrain

## Abstract

The aim of the present study is to evaluate the effects of 60-day artichoke leaf extract (ALE) supplementation (250mg, twice daily) on cytokines levels, natural killer cell (NK) response, and lipo-metabolic profile (HDL, LDL, and total-cholesterol, triglycerides (TG), ApoB, ApoA, lipid accumulation product (LAP), glucose, insulin, and homeostasis model assessment of insulin resistance (HOMA-IR)) in twenty adults (9/11 males/females, age=49.10 ± 13.74 years, and BMI=33.12 ± 5.14 kg/m^2^) with low HDL-C and mild hypercholesterolemia. Hierarchical generalized linear model, adjusted for sex, BMI, and age, has been used to evaluate pre-post treatment changes. A significant increase for HDL-C (*β*=0.14, p=0.0008) and MCP-1 (*β*=144.77, p=0.004) and a significant decrease for ApoB/ApoA (*β*=-0.07, p=0.03), total-C/HDL-C ratio (*β*=-0.58, p<0.001), and NK response at stimulus low (*β*=0.43, p=0.04), medium (*β*=0.40, p<0.001), and high (*β*=0.42, p=0.001) have been found. These results support the benefits of ALE supplementation on metabolic profile.

## 1. Introduction

Recently, a growing level of attention has been devoted to the relationship between inflammation and lipid metabolism, since it has been demonstrated that cytokines may affect both concentration and composition of plasma lipoproteins, including HDL-C [1, 2], by modifying the activity of the triglycerides lipases. Furthermore, it has been shown that proinflammatory cytokines inhibit the activity of lipoprotein lipase (LPL) [1, 3] and enhance the lipolytic activity of endothelial lipase (EL) [4, 5], whose effects are to reduce the levels of HDL-C during acute or chronic inflammatory states. In clinical studies, it has been shown that inflammation is able to affect lipoprotein metabolism [6], which is reflected by a decrease in serum levels of HDL-C in the presence of inflammation. In this regard, Zuliani et al. [7] have shown the existence of an inverse relationship between plasma IL-6 and HDL-C concentrations in community-dwelling older persons.

The relationship between cytokines and HDL-C levels is an important topic to investigate, since low HDL-C level represents one of the most prevalent lipid abnormalities in the subjects affected by coronary heart disease (CHD) [8, 9] and found to be associated with increased incidence of CHD [10, 11]. Furthermore, several recent guidelines [12–14] consider low HDL-C level a strong and independent risk factor for CHD. Currently, there are no commercial drugs that cause an increase in HDL-C. However, scientific literature has shown that there are nutritional supplements containing botanical extracts that have the ability to increase HDL-C. In particular, numerous very recent systematic reviews, meta-analysis, and position papers have demonstrated that artichoke leaf extract (ALE) could represent a potential treatment option for subjects with primary mild hypercholesterolaemia, particularly with low HDL-C level [15–18].

Although the mechanisms explaining the HDL-C increasing effect of ALE are not well known, the most likely explanation could be related to their polyphenolic content and in particular to chlorogenic acid [19–21]. This compound could favour the increase in HDL-C through the enhancement of the activity of paraoxonase-1 (PON-1). PON-1 is an enzyme associated with HDL-C which prevents its oxidation, thus favouring its antioxidative and anti-inflammatory effects [22]. In a vitro study, Gugliucci and Bastos [23] have demonstrated that chlorogenic acid protects PON-1 activity in HDL-C. Previous human studies have proved that the increase in PON-1 activity correlates strongly with an increase in HDL-C [24, 25]. These data lead to hypothesize that chlorogenic acid, present in ALE extract, may induce an increase in PON-1 activity and, as a consequence, may favour the increase in HDL-C in humans.

Given this background, the aim of the present study is to evaluate the effects of 60-day ALE supplementation on baseline cytokines (IL1, IL6, TNF-alpha, and MCP-1) levels, natural killer (NK) response to a low, medium, and high stimulus, and lipo-metabolic profile, such as HDL-C, LDL-C, total-C/HDL-C ratio, TG, ApoB/ApoA ratio, LAP, glucose, insulin, WC, and HOMA-IR, in adults with low HDL-C and mild hypercholesterolemia.

## 2. Materials and Methods

### 2.1. Study Design

This is a one-group pretest-posttest quasi-experimental design, in which all the participants received the ALE treatment, so there is no control group and as a consequence the study is not randomized.

### 2.2. Subjects

The experimental protocol has been approved by the Ethics Committee of the University of Pavia and volunteers have given their written, informed consent. Subjects were recruited from the Outpatient Unit for the treatment of Metabolic Pathologies, Istituto S. Margherita, Azienda di Servizi alla Persona di Pavia, University of Pavia, and have been screened through a procedure involving a clinical assessment, an interview, and the total-C, HDL-C, and LDL-C serum evaluation. Subjects, aged 18–60 years, with body mass index (BMI) ranging between 19 and 30 kg/m2, with low HDL-C (<1,04 mmol/l for men and 1,3 mmol/l for women), mild hypercholesterolaemia (5.4–7.0 mmol/l), and no history of CVD, volunteered for the trial. None of the subjects have ever taken any medication likely to affect lipid metabolism, and all of them were free for overt liver, kidney, and metabolic diseases, such as diabetes and thyroid disease. The screening has excluded subjects who had fasting plasma glucose >7.0 mmol/L, TG ≥ 5.6 mmol/L, smoked, or drank more than two standard alcoholic beverages/day (20 g of alcohol/day) as well as those who were pregnant or lactating, or if they had entered menopause. Subjects have been also excluded if they had taken medications for weight loss. Physical activity has been recorded and patients have been asked to maintain their usual habit throughout the study period.

### 2.3. Anthropometric Measurements

Anthropometric measurements (weight, height, WC, and BMI) have been collected according to standard procedures [26].

### 2.4. Diet and Dietary Supplement

The subjects have followed balanced personalized isocaloric energy diet set up by a senior dietitian. The dietary treatment has been associated with two daily oral assumptions (before lunch and dinner) of film-coated tablets of 250 mg of standardized ALE (≥20% caffeoylquinic acids, ≥5% flavonoids, and ≥5% cynaropicrin). The tablets have been provided by Indena (Milan, Italy). The supplementation period is 60 days. Compliance to the supplementation regimen is defined as the number of tablets actually taken by each subject, divided by the number of tablets that should have been taken over the course of the study.

### 2.5. Biochemical Analyses

Fasting blood samples have been collected between 07.45 and 09.00 am on baseline (t0) and after 60 days (t1) of treatment for the evaluation of LDL-C, total-C, HDL-C, TG, ApoA, ApoB, glucose, insulin, NK activity, and cytokines (IL1-beta, IL6, TNF-alpha, and MCP-1).

In order to avoid venipuncture stress, blood samples have been obtained through an indwelling catheter inserted in an antecubital vein. Blood samples have been immediately centrifuged and the serum (or plasma) was stored at -80°C until assayed. Fasting blood glucose, total-C, LDL-C, HDL-C, and TG levels have been measured by an automatic biochemical analyser (Hitachi 747, Tokyo, Japan). All assays have been carried out on Cobas Mira Plusw (Roche Diagnostic Systems, Welwyn Garden City, UK) equipment using a standard control serum to ensure accuracy of measurements. Finally, for the assessment of safety, routine blood biochemistry parameters (blood cell count, serum protein electrophoresis, serum creatinine, liver, and thyroid function) have been evaluated with routine methods at the beginning and at the end of the intervention.

HOMA-IR has been calculated using the formula (glucose [mmol/L] X insulin [mU/L])/22.5 [27].

LAP has been calculated using the formula proposed by Kahn: for men=WC[cm]-65]x(TG[mmol/L]); for women =WC[cm]-58]x(TG[mmol/L]) [28].

### 2.6. Natural Killer Activity

In order to evaluate NK activity, peripheral blood mononuclear cells (PBMCs) have been isolated from whole heparin blood samples as described by Castellazzi et al. [29]. Briefly, PMBCs have been isolated after a centrifugation on a Ficoll-Hypaque gradient and resuspended in a freezing solution containing Foetal Calf Serum (FCS, Euroclone) and 10% dimethyl sulphoxide (DMSO; Edwards Lifesciences) and cryopreserved at -180°C until assay performing. Cytotoxic activity of NK cells has been evaluated as percentage of lysis of K562 line cells (human cell lymphoma) as described by Valsecchi et al. [30]. Briefly, PBMCs have been thawed and resuspended in RPMI 1640 (Euroclone) + 10% FCS incubated overnight at 37°C, 5%CO_2_. K562 cells have been labelled with ^51^CR (Perkin Elmer) overnight at 37°C, 5%CO_2_. The following day, PBMCs and K562 cells have been cocultured for 4 hours in U bottom 96-well microplates (Corning Costar) and then harvested, and culture supernatants have been transferred in 96-well lumaplates (Perkin Elmer). Target cells (K562) and effectors (PBMCs) have been plated in different concentration ratios, with a fixed number of target cells and a variable number of effectors. The following ratios have been used: 1:10, 1:30, 1:100 (target/effector cells). Radioactivity intensity has been measured using a Top Count Gamma Counter (Perkin Elmer) and is proportional to lysis percentage, consequence of cytotoxic activity of NK cells. NK activity is expressed as lysis percentage of K562 cells induced by PBMCs.

### 2.7. Evaluation of Plasmatic Cytokine Levels

Plasmatic levels of IL1-beta, IL6, TNF-alpha of patients at t0 and t1, have been evaluated using an ELISA test, according to manufacturer instructions, as described by Castellazzi et al. [29]. Briefly, microtiter plates have been coated with purified monoclonal antibody anti-human IL6 (1 ug/ml, Thermo Scientific), IL1-beta, and TNF-alpha (4 ug/ml, R&D system). After stabilization with 2% BSA in PBS for 1 h, diluted samples have been added. Monoclonal biotin labeled antibody anti-human IL6 (200ng/ml, Thermo Scientific) and IL1-beta and TNF-alpha (300 ng/ml and 50 ng/ml, respectively, R&D System) have been used.

The enzymatic reactions have been developed for IL6 with Poly-HRP Streptavidin and TMB solution (Thermo Scientific) and for IL1-beta and TNF-alpha with Streptavidin and A&B Substrate solutions (R&D system). Absorbances have been read at 450nm using Tecan Sunrise (Tecan) microplate reader and data have been analyzed with Magellan software (Tecan). Reproducibility and specificity of the assay have been previously verified.

For the detection of MCP-1, a commercial kit, containing primary and conjugated antibodies, standard and streptavidin, has been used. Briefly, the assays utilize a coating antibody specific for MCP-1 coated on a 96-well microtiter plate. After washing off the excess and unbound materials, the wells have been washed again and a streptavidin-HRP (SPP) conjugate has been added to the antibody-antigen-antibody complex. After another wash, a chromogenic substrate (TMB) has been introduced. The enzymatic reaction has been stopped and absorbance read at 450nm using Tecan Sunrise (Tecan) microplate reader and data analyzed with Magellan software (Tecan). It is not possible to set a normal range of values for these cytokines in the population, because these are not diagnostic routine tests. However, in data sheet of MCP-1, a range of values in healthy adult volunteers between 74 and 760 pg/ml, detected in serum using an experimental ELISA kit, has been reported. The sensibility of each single assay is IL1-beta 0.5-300 pg/ml; IL6 0.3-125 pg/ml; TNF-alpha 1.7-500 pg/ml; MCP-1 1.05-540 pg/ml.

### 2.8. Safety

Safety has been assessed by the measurement of blood pressure and by routine blood biochemistry parameters at t0 and t1 and by recording all the adverse events (AEs), which are based on spontaneous reporting by subjects as well as opened enquiries by members of the research staff.

### 2.9. Statistical Analysis

Missing data have been imputed via iterative stepwise regression imputation using standard and robust methods (*VIM R package*) [31]. Normality has been checked by Shapiro–Wilk test. Continuous variables have been summarized as mean ± standard deviation (SD).

We define as endpoint the difference between the measurements at t0 and t1 (t1-t0)); more specifically we define as primary endpoint that of HDL-C and as secondary endpoints those of LDL-C, VLDL-C, total-C/HDL-C ratio, TG, ApoB/ApoA ratio, LAP, HOMA-IR, glucose, insulin, TG, WC, and NK response to different stimuli. We consider age, sex, and BMI as possible confounders.

For each endpoint, a nonparametric Wilcoxon signed ranks test has been used to assess the differences between the measurements at t0 and t1 (t1-t0). A hierarchical generalized linear model (HGLM) (*HGLM R package*) [32] has been applied to evaluate pre-post treatment changes. A subject random effect (1∣id) has been used to adjust the models for intrasubject variability produced by the two repeated measurements (at t0 and t1) carried out on the same patients (20 patients ×2 measurements = 40 observations but only 20 of them independent). The model parameter is interpreted as change over time. P-values < 0.05 on 2-sided test are considered as statistically significant. To evaluate the effect of treatment on NK response at different stimuli we use the same HGLM as above, also testing the interaction between time and stimuli as a categorical variable. We compared the goodness of fit of the two models (with interaction versus no interaction) via a likelihood ratio test (LRT). Given the small sample size and the low power, a p-value<0.10 for the LRT can be considered as statistically significant; thus we explore the difference between pre-post treatment in each stimulus.

All models are adjusted for sex, BMI, and age. Lastly, pairwise partial correlations, adjusted for sex and age (**z**), between HDL-C (x) and all the secondary endpoints (y), r(x,y∣**z**), were calculated at t0 and t1, in order to understand the relationship between the primary and the secondary endpoints. The data have been analyzed using the R software [33].

## 3. Results

**Figure 1:**
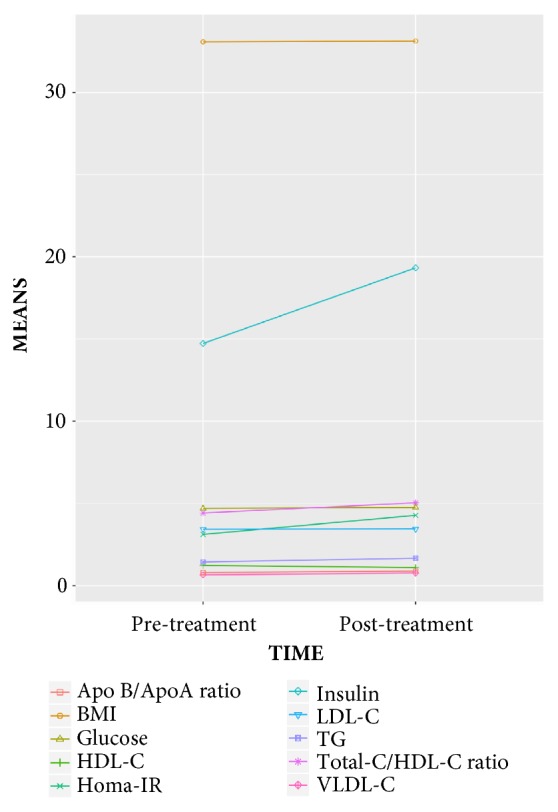
Pre- and posttreatment means.

We have analyzed 20 sedentary, abstemious, and nonsmokers subjects (9 males and 11 females, age = 49.10 ± 13.74 years) with low HDL-C and mild hypercholesterolemia. In Table 1 are reported for each variable its mean and SD (at t0 and t1) and in Figure 1 are plotted the pre- and posttreatment means.

In many individuals, the instrument has been unable to measure the value of the concentration of the IL1, IL6, and TNF-alpha cytokines and hence we cannot evaluate the treatment effect on these cytokines.

Firstly, nonparametric Wilcoxon signed ranks test has shown that the difference between the measurements at t0 and t1 is statistically significant in cytokines levels (MCP-1 [p= 0.002]), in lipo-metabolic profile (HDL-C [p=0.0002], total-C/HDL-C ratio [p=0.0001], ApoB/ApoA ratio [p=0.015], HOMA-IR [p=0.70], LAP [p= 0.14], TG [p=0.12], glucose [p=0.59], insulin [p=0.81], WC [p=0.007]), and in NK responses to a low [p= 0.9854], medium [p= 0.1429], and high [p= 0.114] stimuli.

Secondly, for the primary and for each secondary endpoint we have evaluated pre- and post-treatment change, via HGLM, and the results are reported in Table 2.

To evaluate the effect of treatment on NK response at different stimuli, we first compared the goodness of fit of the two models (with interaction time∗stimuli versus no interaction) via a LRT (p= 0.08); thus we have explored the difference between pre-post treatment in each stimulus. Results are shown in Table 3.

**Figure 2:**
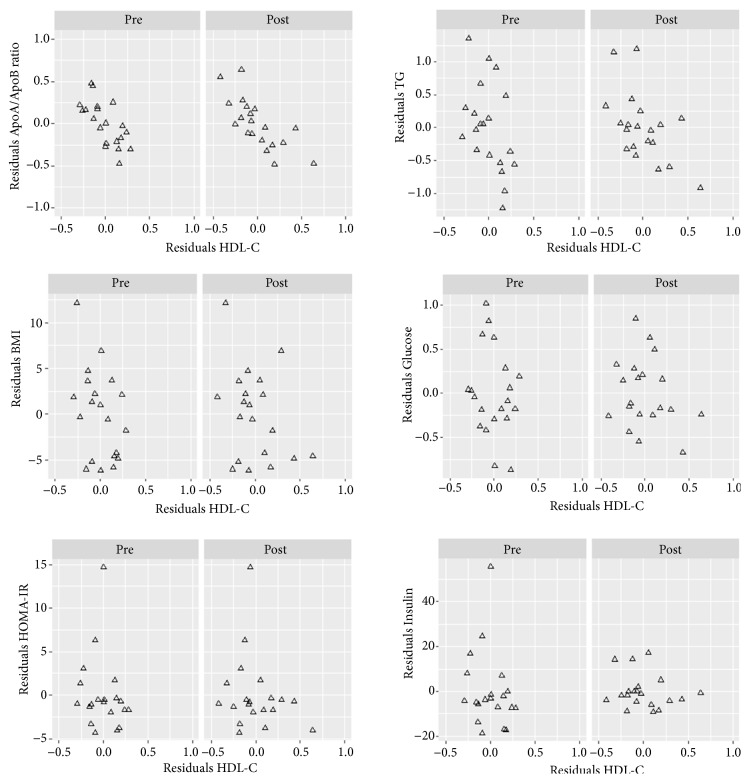
Partial correlation, adjusted for sex and age, between HDL-C and each secondary endpoint before and after treatment.

Lastly, we have assessed the pairwise partial correlations, adjusted for age and sex, between HDL-C and all the secondary endpoints, at t0 (Table 4) and t1 (Table 5). In order to visualize the relationship between the primary and all the secondary endpoints in terms of partial correlation, in Figure 2 we have plotted the residuals of the linear regression between each secondary endpoint as outcome and age and sex as predictors (y-axes) and the residuals of the linear regression between HDL-C as outcome and age and sex as predictors (x-axes).

The plots showing a linear trend refer, as expected, to the significant partial correlations. A significant pretreatment correlation has been found between HDL-C and the following parameters: MCP-1 (r=-0.57, p=0.01), ApoB/ApoA ratio (r=-0.69, p=0.001), and LAP (r=-0.50, p=0.03). A significant posttreatment correlation has been found between HDL-C and the following parameters: ApoB/ApoA ratio (r=-0.75, p=0.0004), LAP (r=-0.53, p=0.02), and TG (r=-0.54, p=0.02).

Concerning safety, no adverse events have been reported.

## 4. Discussion

The main result of this study is that the 60-day supplementation with ALE is associated with a significant increase in HDL-C, MCP-1, and NK response and with a decrease in ApoB/ApoA and total-C/HDL-C ratio in a group of adults with low HDL-C and mild hypercholesterolemia.

This beneficial effect on lipid metabolism is in agreement with numerous very recent systematic reviews, meta-analysis, and position papers indicating ALE as a potential treatment option for subjects with primary mild hypercholesterolaemia, particularly if level of HDL-C is low [15–18], while some previous ALE intervention studies failed to show an increase in HDL-C [34–36]. This different result could be due to the smaller sample size and lower homogeneity of the population studied by Englisch, Lupattelli, and Bundy. Moreover, the different purification and standardization, as well as the stability of the supplements, could play a key role in elucidating the clinical and biological effects of ALE. The efficacy of ALE supplementation on lipid metabolism is related to their polyphenolic content and in particular to chlorogenic acid [19–21] that could favour the increase in HDL-C through the enhancement of the activities of paraoxonase-1 (PON-1). PON-1 is an enzyme associated with HDL which prevents the oxidation of HDL-C, thus favouring its antioxidative and anti-inflammatory effects [22].

In addition to chlorogenic acid, ALE displays high potential as natural source of minerals and other phytochemicals compounds with antioxidant and anti-inflammatory properties [37]. Among these polyphenolic compounds, verbascoside has shown a potential spectrum of many activities including antioxidant and anti-inflammatory [38–41]. Another polyphenolic ALE compound, apigenin-7-glucoside, has been found to block the release of several varieties of enzymes involved in inflammation including especially lipoxygenases and cyclooxygenases [42, 43] leading to inhibition of proinflammatory molecules NFkB activation and of neutrophil infiltration in tissues.

Regarding inflammation, our results show a significant increment of MCP-1 after ALE supplementation. Monocyte chemoattractant protein-1 (MCP-1/CCL2) is one of the key chemokines that regulate migration and infiltration of monocytes/macrophages, required for routine immunological surveillance of tissues, as well as in response to inflammation [44]. In particular, CCL2 expression could be associated with the development of polarized Th_2_ responses [45, 46] and also with the enhancement of the secretion of IL4 by T cells [47, 48]. Moreover, the increase of Th2 cytokines, such as IL4, is able to inhibit Th1 cytokines production, such as IL6 and IFN-gamma that play a crucial role in the development of acute and chronic inflammatory processes and in the activation of innate immune response, in particular the NK activity. ALE supplementation seems to increase plasma levels of MCP-1/CCL2 and contribute to the balance between Th1 and Th2 immune response that is unbalanced towards Th1 in a condition of chronic systemic inflammation.

Our results also show a decrease of NK activity in patients after ALE supplementation. NK are cells of the innate immune system with high antitumor, antiviral, and antimicrobial activity [49]. NK activation can be shaped by inflammasomes [50], which are triggered in response to cell damage and inflammation. Our results could be explained by considering that ALE possess anti-inflammatory properties able to downregulate the activation of inflammasomes in these patients and to silence Th1 inflammatory response. Moreover, these results are in agreement with the observed increase in MCP-1 production that promotes the differentiation of Th2 cells and reduces the Th1 cytokines production, such as IFN-gamma, that have a key role in the activation of NK cells.

Regarding other cytokines evaluated in this study (IL1, IL6, and TNF-alpha), it is not possible to set a normal range of values in our population. The only other two studies [7, 51] that evaluate cytokine levels in subjects with low HDL-C consider only elderly subjects and show that high IL6 plasma levels are associated with low HDL-C levels in community-dwelling older subjects. We hypothesize that the difference in age in our study could be the reason why we could not detect IL1, IL6, and TNF-alpha.

To prevent weight loss from influencing the results of the study, really significant situation specially for metabolic results, we asked patients to follow an isocaloric personalized diet that was individually prepared by a senior dietitian. During the study, the weight of the patients remained stable, so we exclude the effects of a body weight loss on our results. In addition, all models are adjusted for sex, BMI, and age.

A limitation of our study is given by the use of a one-group pretest-posttest quasi–experimental design to investigate the effect of 60-day supplementation with ALE on NK response and lipo-metabolic profile in a group of adults with low HDL-C and mild hypercholesterolemia. This study design lacks control group and hence there are threats to internal validity (e.g., regression to the mean). Such study does not allow drawing causal conclusions on the effect of the treatment on the outcomes analyzed; this means that other factors could influence the outcomes other than the investigated treatment [52, 53]. The choice of this kind of study was driven by the small sample size availability and by the practical necessities imposed by limited funding.

Despite the limitations reported above, this study, less expensive and time consuming than randomized controlled trials (RCTs), offers the opportunity to compare the difference between pre- and posttreatment measurements, thus investigating potential effect of ALE. Nevertheless, given the short interval (i.e., 60 days) between pre- and postmeasurements the internal validity could be considered as safeguarded, in the sense that the within-subject variation due to chance might be limited.

## 5. Conclusions

Despite the limitation given by the use of a one-group pretest-posttest quasi–experimental design, which we discussed above, the strength of our study relies in the evaluation, for the first time in the literature, of cytokine levels and NK activity in a specific population group: adult subjects with low HDL-C and moderate hypercholesterolemia. Furthermore, our study confirms the positive action of ALE in increasing HDL-C and in decreasing ApoB/ApoA and total-C/HDL-C ratio. These results prompt further our experimental study with a randomized control group to evaluate the causal effect of ALE on NK responses and lipo-metabolic profile and to investigate the correlation between inflammation and lipid metabolism in adult subjects with low HDL-C and moderate hypercholesterolemia.

## Figures and Tables

**Table 1:** Descriptive statistics of the sample at baseline (t0) and after 60 days of treatment (t1).

**N= 20 (9 males)**	**Baseline(t0)**	**After 60 days (t1)**
	**Mean ± SD**	**Mean ± SD**
MCP-1 (pg/ml)	509.9 ± 213.08	654.7 ± 248.84
HDL-C (mmol/L)	1.10 ± 0.21	1.23 ± 0.28
VLDL-C (mmol/L)	0.77 ± 0.36	0.66 ± 0.25
LDL-C (mmol/L)	3.45 ± 0.83	3.43 ± 0.84
Total-C/HDL-Cr	5.04 ± 1.53	4.42 ± 1.40
NK activity 1:10 (% lysis)	8.44 ± 6.27	9.07 ± 7.74
NK activity 1:30 (% lysis)	15.93 ± 10.43	12.78 ± 8.10
NK activity 1:100 (% lysis)	30.90 ± 13.50	26.75 ± 11.33
TG (mmol/L)	1.67 ± 0.78	1.44 ± 0.55
Glucose (mmol/L)	4.76 ± 0.58	4.70 ± 0.41
Insulin (mU/L)	19.33 ± 18.27	14.73 ± 8.01
WC (cm)	108.65 ± 15.05	107.75 ± 15.25
HOMA-IR	4.28 ± 4.58	3.12 ± 1.84
LAP	81.31 ± 49.03	70.48 ± 44.11
ApoB/ApoA ratio	0.89 ± 0.29	0.80 ± 0.32
BMI (kg/m^2^)	33.12 ± 5.14	33.07 ± 5.08

**Table 2:** Estimate, standard error, and p-value of the treatment effect on the primary and secondary endpoints, evaluated as difference between pre-post treatment, using HGLM.

**Primary/Secondary Endpoints**	**Estimate**	**Stand. Error**	**p-value**
MCP-1	144.77	44.77	0.004
HDL-C	0.14	0.04	0.0008
Total -C/HDL-C ratio	-0.58	0.12	8.5e-05
TG	-0.23	0.12	0.07
ApoB/ApoA ratio	-0.07	0.03	0.03
HOMA-IR	-0.99	0.94	0.30
LAP	-10.895	5.8037	0.0744
VLDL-C	-0.11	0.06	0.07
LDL-C	0.03	0.10	0.76
Glucose	-0.08	0.10	0.40
Insulin	-3.69	3.63	0.32
WC	-0.9000	0.3092	0.00897

All models are adjusted for sex, BMI, and age. In bold significant results (P<0.05).

**Table 3:** Estimate, standard error, and p-value of the treatment effect on **NK responses at different stimuli**, evaluated as difference between pre-post treatment, using HGLM.

**NK response**	**Estimate**	**Stand. Error**	**p-value**
Low stimulus	0.43	0.32	**0.04**
Medium stimulus	0.40	0.16	**5.2 e-04**
High stimulus	0.42	0.16	**0.001**

All models are adjusted for sex, BMI, and age. In bold significant results (P<0.05).

**Table 4:** Partial correlation between HDL-C and the variables under study before treatment.

**Variables**	**r(x,y**∣**z)**	**95**%**CI**	**p-value**
MCP-1	-0.57	-0.82,0.14	**0.01**
ApoB/ApoA ratio	-0.69	-0.88,-0.33	**0.001**
HOMA-IR	-0.17	-0.59,0.31	0.49
LAP	-0.50	-0.78,-0.04	**0.03**
NK low stimulus	0.10	-0.38,0.54	0.69
NK medium stimulus	0.04	-0.44,0.49	0.89
NK high stimulus	0.20	-0.29,0.61	0.42
BMI	-0.40	-0.73,0.08	0.10
TG	-0.45	-0.76,0.02	0.06
Insulin	-0.19	-0.61,0.29	0.43
Glucose	-0.19	-0.60,0.31	0.46
WC	-0.34	-0.70,0.14	0.16

In bold significant results (P<0.05).

**Table 5:** Partial correlation between HDL-C and the variables under study after treatment.

**Variables **	**r(x,y**∣**z)**	**95**%**CI**	**p-value**
MCP-1	-0.43	-0.74,0.04	0.07
ApoB/ApoA ratio	-0.75	-0.89,-0.42	**0.0004**
HOMA-IR	-0.18	-0.59,0.31	0.47
LAP	-0.53	-0.80,-0.08	**0.02**
NK low stimulus	0.39	-0.09,0.72	0.11
NK medium stimulus	-0.05	-0.50,0.43	0.84
NK high stimulus	-0.03	-0.49,0.44	0.89
BMI	-0.32	-0.68,0.17	0.19
TG	-0.54	-0.81,-0.11	**0.02**
Insulin	-0.17	-0.59,0.32	0.49
Glucose	-0.22	-0.62,0.27	0.37
WC	-0.38	-0.72,0.10	0.11

In bold significant results (P<0.05).

## Data Availability

The hematochemical and statistical data used to support the findings of this study are included within the article.
